# Dimethyl Fumarate Blocks Tumor Necrosis Factor-Alpha-Driven Inflammation and Metabolic Rewiring in the Retinal Pigment Epithelium

**DOI:** 10.3389/fnmol.2022.896786

**Published:** 2022-06-23

**Authors:** Daisy Y. Shu, Scott I. Frank, Tessa C. Fitch, Margarete M. Karg, Erik R. Butcher, Emmanuella Nnuji-John, Leo A. Kim, Magali Saint-Geniez

**Affiliations:** ^1^Schepens Eye Research Institute of Massachusetts Eye and Ear, Boston, MA, United States; ^2^Department of Ophthalmology, Harvard Medical School, Boston, MA, United States; ^3^Cold Spring Harbor Laboratory, School of Biological Sciences, Cold Spring Harbor, NY, United States

**Keywords:** inflammation, metabolism, mitochondria, retinal pigment epithelium, tumor necrosis factor-alpha, oxidative phosphorylation, glycolysis, age-related macular degeneration

## Abstract

The retinal pigment epithelium (RPE) acts as a metabolic gatekeeper between photoreceptors and the choroidal vasculature to maintain retinal function. RPE dysfunction is a key feature of age-related macular degeneration (AMD), the leading cause of blindness in developed countries. Inflammation is a key pathogenic mechanism in AMD and tumor necrosis factor-alpha (TNFα) has been implicated as a pro-inflammatory cytokine involved in AMD. While mitochondrial dysfunction has been implicated in AMD pathogenesis, the interplay between inflammation and cellular metabolism remains elusive. The present study explores how the pro-inflammatory cytokine, TNFα, impacts mitochondrial morphology and metabolic function in RPE. Matured human primary RPE (H-RPE) were treated with TNFα (10 ng/ml) for up to 5 days. TNFα-induced upregulation of IL-6 secretion and inflammatory genes (IL-6, IL-8, MCP-1) was accompanied by increased oxidative phosphorylation (OXPHOS) and reduced glycolysis, leading to an increase in cellular adenosine triphosphate (ATP) content. Transmission electron microscopy (TEM) revealed defects in mitochondrial morphology with engorged mitochondria and loss of cristae integrity following TNFα treatment. Pre-treatment with the anti-inflammatory drug, 80 μM dimethyl fumarate (DMFu), blocked TNFα-induced inflammatory activation of RPE (IL-6, IL-8, MCP-1, CFH, CFB, C3) and normalized their bioenergetic profile to control levels by regulating PFKFB3 and PKM2 gene expression. Furthermore, DMFu prevented TNFα-induced mitochondrial dysfunction and morphological anomalies. Thus, our results indicate that DMFu serves as a novel therapeutic avenue for combating inflammatory activation and metabolic dysfunction of RPE in AMD.

## Introduction

Dysfunction of retinal pigment epithelial cells (RPE) is an early sign of AMD and loss of RPE integrity increases as AMD progresses (Bonilha, 2008; Ao et al., 2018). Inflammation is a key pathogenic mechanism in AMD (Datta et al., 2017) through complement system activation, upregulation of inflammatory cytokines and recruitment of macrophages to the outer surface of the RPE-Bruch’s membrane interface in AMD eyes (Hageman et al., 2001) that facilitates drusen growth (Levy et al., 2015). A critical cytokine mediating inflammatory responses throughout the body is tumor necrosis factor-alpha (TNFα), which has been implicated in the pathogenesis of AMD (Oh et al., 1999; Wan et al., 2010; Kutty et al., 2016). Single nucleotide polymorphisms (SNPs) have been detected in the TNFα gene in AMD patients (Wan et al., 2010; Chernykh et al., 2019) and TNFα decreases the expression of genes regulating RPE function, including RPE65 (Kutty et al., 2016). Further, lower serum levels of TNFα have been associated with improved visual acuity after anti-VEGF therapy in wet AMD patients (Khan et al., 2021), highlighting TNFα as a pathogenic driver of wet AMD.

Defects in RPE mitochondria and metabolism drive AMD pathogenesis (Fisher and Ferrington, 2018; Kaarniranta et al., 2020; Zhang et al., 2020). Work in our laboratory has further implicated metabolic and mitochondrial dysfunction of RPE in AMD (Iacovelli et al., 2016; Satish et al., 2018; Rosales et al., 2019; Shu et al., 2020, 2021). Despite evidence for both inflammation and metabolic dysfunction in AMD, there is a paucity of literature dissecting the crosstalk between these two mechanisms in the AMD field. Hence, this study sought to examine whether TNFα-driven inflammation in RPE is accompanied by changes in mitochondria and metabolism. Further, we investigated the efficacy of the fumaric acid ester, dimethyl fumarate (DMFu), in blocking TNFα-driven inflammation and metabolic changes in RPE.

Fumaric acid esters have been used as anti-inflammatory treatments for the autoimmune disease, psoriasis, for over 20 years (Blair, 2018). DMFu is FDA-approved for the treatment of relapsing multiple sclerosis in the form of delay-release capsules for oral use (Cada et al., 2013). DMFu is rapidly hydrolyzed to its active metabolite, monomethyl fumarate (MMF), which has a terminal half-life of 1 h. MMF is then metabolized by the TCA cycle to fumaric acid, citric acid, glucose, and carbon dioxide. The potent anti-inflammatory effect of DMFu is linked to its ability to block the NF-κB pathway (Borgers et al., 2001), a downstream target of TNFα activation.

Here, we bridge the gap between inflammation and metabolic dysfunction in RPE showing that the pro-inflammatory effects of TNFα are associated with a dramatic change in mitochondrial morphology, function, and rewiring of the metabolic pathways. We reveal that pre-treatment of primary human RPE with the metabolic drug, DMFu, blocks the pro-inflammatory effects of TNFα, further highlighting the intertwined link between inflammation and metabolism.

## Materials and Methods

### Cell Culture

Primary human fetal retinal pigment epithelial cells (H-RPE, Lonza Cat #00194987) were cultured in RtEGM Retinal Pigment Epithelial Cell Growth Medium supplemented with RtEGM SingleQuots (Lonza, Walkersville, MD, United States) as described previously (Shu et al., 2021). H-RPE were maintained at 37°C and 5% CO_2_ in a humidified incubator and passaged 1:3 at a maximum of 5 passages. Half media changes for H-RPE were performed every 2–4 days for a month to ensure proper RPE maturation and pigment accumulation. Cells were serum starved for 2–3 days before treatment for up to 5 days with recombinant human TNFα (Peprotech, Rocky Hill, NJ, United States) at 10 ng/mL and/or 80 μM dimethyl fumarate (242926, Sigma, St Louis, MO, United States) dissolved in the vehicle control, DMSO at the equivalent concentration of 0.04% (D4540, Sigma, St Louis, MO, United States). Brightfield images were captured on the EVOS M7000 Imaging System (Invitrogen, ThermoFisher, Waltham, MA, United States). Cells were tested monthly for mycoplasma contamination using the Mycoplasma PCR test (Applied Biological Materials, Cat #G238).

### Quantitative PCR

Quantitative PCR (qPCR) assays were performed as previously described (Shu et al., 2021). Briefly, the E.Z.N.A. total RNA Kit I (Omega BioTek, Norcross, GA, United States) was used to extract total RNA and concentrations were determined using the NanoDrop Spectrophotometer ND-1000 (ThermoFisher Scientific, San Jose, CA, United States). cDNA synthesis was performed using the SuperScript IV VILO MasterMix and the Techne TC-512 Thermal Cycler at 25°C, 10 min; 50°C, 10 min; 85°C, 5 min (ThermoFisher Scientific, San Jose, CA, United States) and amplified by real-time PCR using the PowerUp SYBR Green Master Mix (ThermoFisher Scientific, San Jose, CA, United States) in a LightCycler 480 (Roche, Basel, Switzerland). Reactions were run in duplicate under the following thermal cycling conditions: 50°C, 2 min; 95°C, 2 min, followed by 40 cycles of 95°C for 15 s and 60°C for 1 min. Melt curve analysis was performed to confirm amplification specificity. *Ct* values were normalized to the housekeeping gene, TATA-binding protein (TBP), using the second derivative maximum method. Primer sequences are listed in Table 1.

**TABLE 1:** Primer sequences for qPCR.

Gene symbol	Gene name	Forward sequence (5′-3′)	Reverse sequence (5′-3′)
*ATP5O*	ATP synthase subunit O	TTTGAATCCCTATGTGAAGCGTT	CCTTGGGTATTGCTTAATCGACC
*C3*	Complement component 3	ACGGCCTTTGTTCTCATCTC	CAAGGAAGTCTCCTGCTTTAGT
*CFB*	Complement factor B	GCTGTGAGAGAGATGCTCAATA	GACTCACTCCAGTACAAAG
*CFH*	Complement factor H	CTGATCGCAAGAAAGACCAGTA	TGGTAGCACTGAACGGAATTAG
*IL-6*	Interleukin-6	ACTCACCTCTTCAGAACGAATTG	CCATCTTTGGAAGGTTCAGGTTG
*IL-8*	Interleukin-8	TTTTGCCAAGGAGTGCTAAAGA	AACCCTCTGCACCCAGTTTTC
*MCP-1*	Monocyte chemoattractant protein-1	CAGCCAGATGCAATCAATGCC	TGGAATCCTGAACCCACTTCT
*PFKFB3*	6-phosphofructo-2-kinase/fructose-2,6-biphosphatase 3	CAGTTGTGCCTCCAATATC	GGCTTCATAGCAACTGATCC
*PGC-1A*	Peroxisome proliferator-activated receptor gamma, coactivator 1 alpha (PPARGC1A)	GTCACCACCCAAATCCTTAT	ATCTACTGCCTGGAGACCTT
*PKM2*	Pyruvate kinase muscle isozyme M2	CAAAGGACCTCAGCAGCCATGTC	GGGAAGCTGGGCCAATGGTACAGA
*VEGFA*	Vascular endothelial growth factor A	GGGCAGAATCATCACGAAGTG	ATTGGATGGCAGTAGCTGCG
*VEGFC*	Vascular endothelial growth factor C	GCCAATCACACTTCCTGCCGAT	AGGTCTTGTTCGCTGCCTGACA
*TBP*	TATA-binding protein	TGCACAGGAGCCAAGAGTGAA	CACATCACAGCTCCCCACCA

### High-Resolution Respirometry

The Seahorse XFe96 Analyzer (Agilent Technologies, Santa Clara, CA, United States) was used to determine real-time bioenergetic profiles. 20,000 H-RPE cells were seeded per well according to the manufacturer’s instructions. For the Mito Stress Test, the medium was replaced with the assay medium (Seahorse XF Base Medium without Phenol Red, Agilent) supplemented with 2 mM GlutaMAX (ThermoFisher), 1 mM pyruvate (Gibco, Carlsbad, CA, United States) and 25 mM glucose (Sigma, St. Louis, MO, United States), pH 7.4 and placed in a 37°C, CO_2_-free, humidified incubator for 1 h. The drug injections were oligomycin (2.5 μM), BAM15 (10 μM) and a combination of rotenone and antimycin A (both at 2 μM). The same reagents were used for the ATP Rate Assay with the BAM15 drug injection excluded. For the Glycolytic Stress Test, the medium was replaced with the assay medium (Seahorse XF Base Medium without Phenol Red, Agilent) supplemented with 1 mM GlutaMAX (ThermoFisher), pH 7.4, and placed in a 37°C, CO_2_-free, humidified incubator for 1 h. The drug injections were glucose (10 mM), oligomycin (2 μM), and 2DG (50 mM). On completion of the Seahorse assays, cells were lysed in cold 1× Cell Lysis Buffer (Cell Signaling Technology, Beverly, MA, United States) supplemented with 1 mM PMSF (Sigma, St Louis, MO, United States) and stored at −80°C. Protein concentration was quantified using the Pierce BCA Assay kit (ThermoFisher, Waltham, MA, United States). Data were normalized to protein content using the XF Wave software by exporting the XF Mito Stress Test, XF Glycolytic Stress Test and XF ATP Rate Assay Test Report Generators to Excel and GraphPad Prism.

Oxygen Consumption Rate (OCR) was also measured using the Resipher (Lucid Scientific, Atlanta, GA, United States). 50,000 H-RPE cells were seeded into the Falcon flat-bottom 96-well microplate (Corning #353072) in H-RPE culture medium with the Resipher oxygen sensing lid. Cells were serum starved for 2 days before treatment with TNFα at 10 ng/ml. Real-time continuous OCR measurements were monitored in the incubator for a week. Data were analyzed using the Resipher web application and Python.

### Measurement of Intracellular ATP Content

ATP content was measured using the ATP bioluminescence assay kit CLS II (Roche, Heidelberg, Germany) as previously described (Shu et al., 2021). Protein content was measured using the Pierce BCA Assay kit. 20 μg of protein was run in duplicate in a white, flat-bottom 96-well microplate. Determination of free ATP was performed against an ATP standard curve. Luminescence was determined at 60 ms integration using a Synergy H1 Plate Reader (BioTek, Winooski, VT, United States).

### Quantification of Mitochondrial Copy Number

Genomic DNA (gDNA) was isolated using the Universal Genomic DNA Kit (CWBio #CW2298S). DNA concentration and purity was measured using the NanoDrop Spectrophotometer ND-1000. 10 ng of gDNA was used for the qPCR reactions and performed as previously described (Shu et al., 2021) using the Clontech Human Mitochondrial DNA (mtDNA) Monitoring Primer Set (Takara Bio Inc., Kusatsu, Shiga, Japan). mtDNA copy number was calculated as the average of the ratio of mtDNA to nuclear DNA using the two pairs of genes mt-*ND5* with *SERPINA1* and *ND1* with *SLCO2B1*.

### Transmission Electron Microscopy

Cells were fixed with quarter strength Karnovsky’s fixative (1% formaldehyde + 1.25% glutaraldehyde, in 0.1 M sodium cacodylate buffer, pH 7.4) for 6 h at room temperature, then transferred into 0.1 M sodium cacodylate buffer, post-fixed with 1% osmium tetroxide in 0.1 M sodium cacodylate buffer for 1 h, en bloc stained with 1% gadolinium triacetate in 0.05 M sodium maleate buffer, 4–6°C, pH 6 for 30 min. Samples were dehydrated with graded ethyl alcohol solutions, transitioned with propylene oxide and infiltrated in tEPON-812 epoxy resin (Tousimis, Rockville, MD, United States) utilizing an automated EMS Lynx 2 EM tissue processor (Electron Microscopy Sciences, Hatfield, PA, United States). The processed samples were oriented into tEPON-812 epoxy resin inside flat molds and polymerized using an oven set at 60°C. The polymerized blocks were cleaved from the coverglass through brief exposure in liquid nitrogen. Semi-thin sections were cut at 1 μm thickness and stained with 1% toluidine blue in 1% sodium tetraborate aqueous solution for assessment by light microscopy. A region containing a monolayer of cells was selected from each sample from the semi-thin toluidine blue stained sections and block face trimmed to <1 mm × 0.5 mm for ultramicrotomy. Ultrathin sections (80 nm) were cut from each block face using a Leica EM UC7 ultramicrotome (Leica Microsystems, Buffalo Grove, IL, United States) and diamond knives (Diatome, Hatfield, PA, United States), to collect onto 2 × 1 mm, single slot formvar-carbon coated grids and 200 mesh copper/rhodium uncoated grids (Electron Microscopy Sciences, Hatfield, PA, United States). The ultrathin sections on grids were stained with aqueous 2.5% gadolinium triacetate and modified Sato’s lead citrate using a modified Hiraoka grid staining system (Seifert, 2017). Grids were imaged using a FEI Tecnai G2 Spirit transmission electron microscope (FEI, Hillsboro, OR, United States) at 80 kV interfaced with an AMT XR41 digital CCD camera (Advanced Microscopy Techniques, Woburn, MA, United States) for digital TIFF file image acquisition. All TEM digital images were captured at 2k × 2k pixels at 16-bit resolution with the same magnification. Images were processed using Contrast Limited AHE (CLAHE) on ImageJ to reduce background noise and amplify mitochondrial ultrastructural features. Images were further analyzed on ImageJ using the hand-trace tool to obtain the area of the mitochondria.

### Statistical Analysis

Statistical analyses were performed using GraphPad Prism 9.1.1. Parametric data were analyzed by one-way ANOVA with Tukey’s *post hoc* analysis or unpaired Student’s *t*-test. Statistical significance was considered when *p* ≤ 0.05 and all data are shown as mean ± SEM. * *p* ≤ 0.05, ^**^ *p* ≤ 0.01, ^***^ *p* ≤ 0.001, and ^****^ *p* ≤ 0.0001.

## Results

### Tumor Necrosis Factor-Alpha-Induced Inflammation in Retinal Pigment Epithelial Cells Is Accompanied by Mitochondrial Dysfunction

**FIGURE 1:**
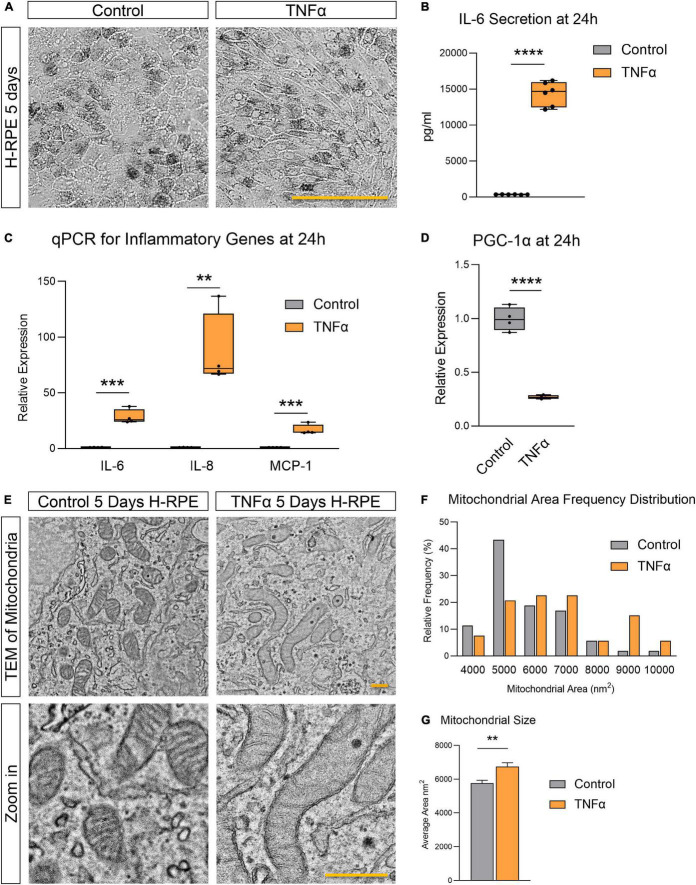
Tumor necrosis factor-alpha-induced inflammation in H-RPE is accompanied by mitochondrial dysfunction. **(A)** Brightfield images of H-RPE cells with and without TNFα treatment. **(B)** IL-6 secretion at 24 h measured using ELISA for H-RPE. **(C,D)** qPCR analysis of inflammatory **(C)** and PGC-1α **(D)** gene expression in H-RPE following 24 h treatment with TNFα. **(E)** TEM images of H-RPE comparing untreated control and TNFα-treatment for 5 days. **(F)** Quantification of mitochondrial size from TEM images presented as a frequency distribution histogram, and **(G)** quantification of average mitochondrial size (unpaired *t*-test). Error bars are means ± SEM. ** *p* ≤ 0.01; *** *p* ≤ 0.001; **** *p* ≤ 0.0001; ns, not significant. Scale bar for panel **(A)** is 100 μm and panel **(E)** is 500 nm.

TNFα induced H-RPE elongation after 5 days of treatment (Figure 1A) while triggering a rapid inflammatory response with increased IL-6 secretion (Figure 1B) and increased gene expression of pro-inflammatory cytokines (IL-6, IL-8, and MCP-1) at 24 h (Figure 1C). This pro-inflammatory activation of RPE by TNFα was accompanied by the repression of PGC-1α, a master regulator of RPE mitochondrial function and homeostasis, at 24 h (Figure 1D). Ultrastructural analysis showed that TNFα induced a dramatic enlargement of mitochondria and cristae sparsity at 5 days as observed by TEM imaging (Figure 1E). Quantification of mitochondrial area showed that TNFα-treated cells displayed a higher frequency of larger mitochondria (Figure 1F) and a significantly larger average mitochondrial area (Figure 1G).

### Tumor Necrosis Factor-Alpha Increased Mitochondrial Respiration and Reduced Glycolysis in Retinal Pigment Epithelial Cells

**FIGURE 2:**
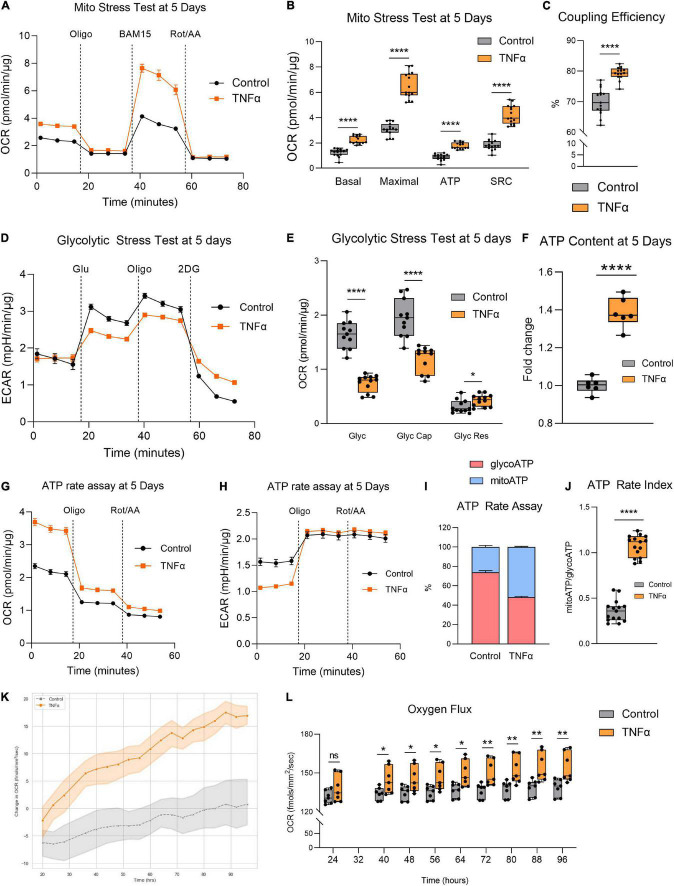
Tumor necrosis factor-alpha increased mitochondrial respiration and reduced glycolysis in RPE. **(A)** Real-time measurement of oxygen consumption rate (OCR) using the Seahorse XFe96 BioAnalyzer to assess **(B)** OXPHOS parameters: basal respiration, ATP-linked respiration, proton leak, spare respiratory capacity (SRC), and maximal respiration based on responses to drug injections of oligomycin (Oligo), BAM15, and rotenone and antimycin A (Rot/AA) at 5 days with and without TNFα (*n* = 10–12, unpaired *t*-test) and **(C)** coupling efficiency. **(D)** Real-time measurement of extracellular acidification rate (ECAR) using the Seahorse XFe96 BioAnalyzer to assess glycolytic function parameters: **(E)** glycolysis, glycolytic capacity, and glycolytic reserve based on responses to drug injections of glucose (Glu), oligomycin (Oligo), and 2-deoxyglucose (2DG) at 5 days of treatment with and without TNFα (*n* = 10–12, unpaired *t*-test). **(F)** Intracellular ATP content at 5 days with or without TNFα (*n* = 6, unpaired *t*-test). **(G)** OCR curves and **(H)** ECAR curves for the Seahorse ATP rate assay at 5 days with TNFα. **(I)** Proportion of mitochondria-derived ATP production and glycolysis-derived ATP production and **(J)** increased ATP Rate Index representing a more oxidative and less glycolytic phenotype with TNFα treatment. **(K)** Real-time monitoring of OCR using the Resipher technology over 96 h following treatment with TNFα and **(L)** quantification of OCR over time (*n* = 7, unpaired *t*-test). Error bars are means ± SEM. * *p* ≤ 0.05; ** *p* ≤ 0.01; **** *p* ≤ 0.0001; ns, not significant.

Given the aberrant mitochondrial morphology observed, we next investigated whether TNFα induced any changes in mitochondrial function by assessing RPE bioenergetics on the Seahorse XFe96 Mito Stress Test. Enlarged mitochondrial size typically confers enhanced bioenergetic capacity (Westermann, 2012). Accordingly, the larger mitochondria observed with TNFα was associated with a significantly increased basal respiration, maximal respiration, ATP production, and spare respiratory capacity (SRC) at 5 days (Figures 2A,B). TNFα also significantly increased coupling efficiency (Figure 2C). In contrast, TNFα reduced glycolysis and glycolytic capacity with a slight elevation of glycolytic reserve (Figures 2D,E). Consistent with the fact that OXPHOS is far more efficient at generating ATP compared to glycolysis, the increased mitochondrial respiration with TNFα was accompanied by a significant increase in intracellular ATP content at 5 days (Figure 2F). Furthermore, the Seahorse ATP rate assay confirmed the enhanced OXPHOS with TNFα, highlighting that a higher proportion of ATP generation was derived from mitochondrial respiration than glycolysis (Figures 2G,H), further indicated by the significantly higher ATP rate index (Figures 2I,J). Continuous monitoring of oxygen flux of H-RPE in the incubator using the Resipher confirmed the elevated OCR with TNFα (Figure 2K) with increasingly significant OCR elevations over time from 24 to 96 h (Figure 2L).

### Pre-treatment With Dimethyl Fumarate Blocks Tumor Necrosis Factor-Alpha-Induced Inflammation and Restores Mitochondrial Health

**FIGURE 3:**
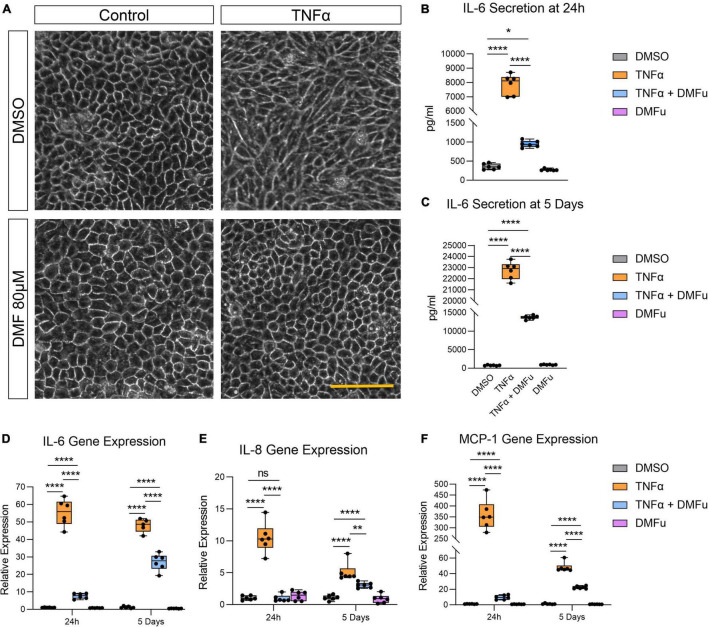
Pre-treatment with dimethyl fumarate (DMFu) blocks TNFα-induced inflammation. **(A)** Brightfield images of H-RPE cells treated with TNFα (10 ng/ml) and/or DMFu (80 μM) or vehicle control (DMSO). **(B,C)** Quantification of secreted IL-6 following TNFα and/or DMFu treatment for **(B)** 24 h and **(C)** 5 days. **(D–F)** qPCR analysis of inflammatory gene expression following treatments on H-RPE for **(D)** IL-6, **(E)** IL-8, and **(F)** MCP-1. *n* = 6, one-way ANOVA with Tukey’s *post hoc* analysis. Error bars are means ± SEM. * *p* ≤ 0.05; ** *p* ≤ 0.01; **** *p* ≤ 0.0001; ns, not significant. Scale bar is 100 μm.

Pre-treatment with DMFu for 2 h prior to TNFα exposure blocked TNFα-induced cellular elongation, maintaining the regular cobblestone-like epithelial morphology (Figure 3A). DMFu alone did not affect cellular morphology (Figure 3A). DMFu significantly suppressed TNFα-induced IL-6 secretion at both 24 h (Figure 3B) and 5 days (Figure 3C). Moreover, DMFu reduced gene expression of inflammatory cytokines including IL-6 (Figure 3D), IL-8 (Figure 3E), and MCP-1 (Figure 3F) at 24 h and 5 days.

**FIGURE 4:**
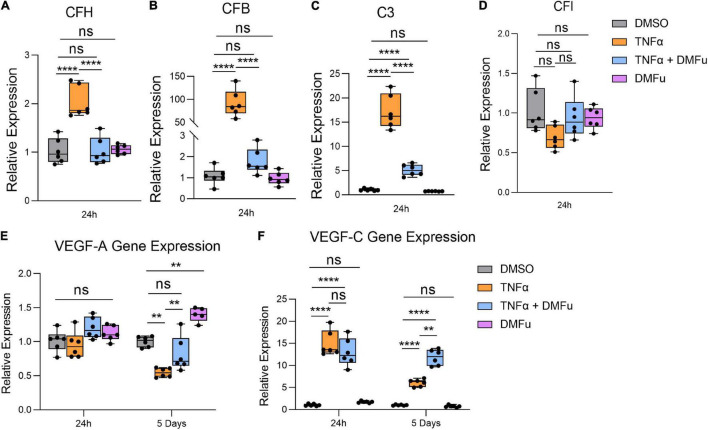
Pre-treatment with dimethyl fumarate (DMFu) blocks TNFα-induced complement activation. qPCR analysis of gene expression of the complement cascade following treatments on H-RPE for **(A)** complement factor H, **(B)** complement factor B, **(C)** complement component 3, **(D)** complement factor I at 24 h. qPCR analysis of gene expression of **(E)** VEGF-A and **(F)** VEGF-C *n* = 5–6, one-way ANOVA with Tukey’s *post hoc* analysis. Error bars are means ± SEM. ** *p* ≤ 0.01; **** *p* ≤ 0.0001; ns, not significant.

Local activation and induction of the alternative complement cascade, an integral part of innate immunity, has been implicated in AMD pathogenesis (Johnson et al., 2001). At 24 h, TNFα robustly enhanced the gene expression of key components of the alternative complement cascade including complement factor H (CFH, Figure 4A), complement factor B (CFB, Figure 4B), and complement component 3 (C3, Figure 4C), which were all significantly suppressed with DMFu pre-treatment. There were no significant changes observed for complement factor I (CFI, Figure 4D).

A critical driver of late-stage neovascular AMD is the pro-angiogenic factor, vascular endothelial growth factor (VEGF) (Rosenfeld et al., 2006). Polarized VEGF expression is important for RPE homeostasis and survival (Ford et al., 2011). Specifically, VEGF-A expression by RPE is essential for choriocapillaris maintenance, and RPE dedifferentiation is associated with loss of VEGF secretion and choriocapillaris dropout (Blaauwgeers et al., 1999; Saint-Geniez et al., 2009). TNFα significantly reduced VEGF-A gene expression at 5 days while pre-treatment with DMFu maintained basal VEGF-A levels (Figure 4E). No significant changes for VEGF-A occurred at 24 h (Figure 4E). VEGF-C is a potent enhancer of vascular permeability and has been identified in RPE derived from choroidal neovascular (CNV) specimens from patients (Martin et al., 2004). VEGF-C gene expression was increased with TNFα at both 24 h and 5 days and appeared to be further increased in the presence of DMFu (Figure 4F).

**FIGURE 5:**
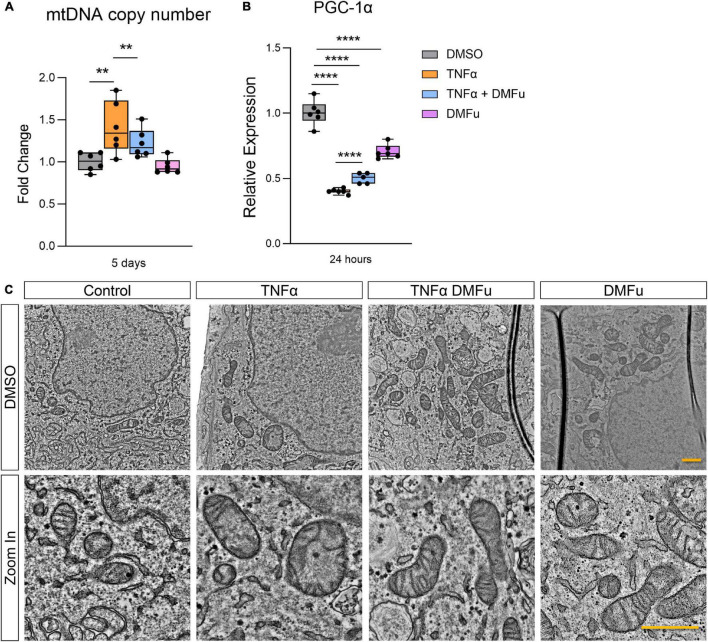
Pre-treatment with dimethyl fumarate (DMFu) blocks TNFα-induced mitochondrial dysfunction. **(A)** Quantification of mtDNA copy number following treatments with TNFα and/or DMFu or the vehicle control (DMSO). **(B)** qPCR analysis of gene expression of PGC-1α on H-RPE following treatments. *n* = 6, one-way ANOVA with Tukey’s *post hoc* analysis. Error bars are means ± SEM. ** *p* ≤ 0.01; **** *p* ≤ 0.0001. **(C)** TEM images of H-RPE treated with DMSO, TNFα 10 ng/ml, TNFα with DMFu, and DMFu alone and magnified images. Scale bar is 1 μm.

The anti-inflammatory activity of DMFu was accompanied by a shift toward normalized mitochondrial function. While TNFα increased mtDNA copy number, co-treatment with TNFα and DMFu reduced mtDNA copy number down to basal levels (Figure 5A). Similarly, co-treatment with TNFα and DMFu significantly enhanced PGC-1α levels compared to TNFα alone; however, this was not sufficient to maintain basal levels of PGC-1α (Figure 5B). On an ultrastructural level, DMFu blocked TNFα-induced loss of cristae architecture and maintained normal mitochondrial size (Figure 5C).

### Pre-treatment With Dimethyl Fumarate Blocks Tumor Necrosis Factor-Alpha-Induced Metabolic Reprogramming

**FIGURE 6:**
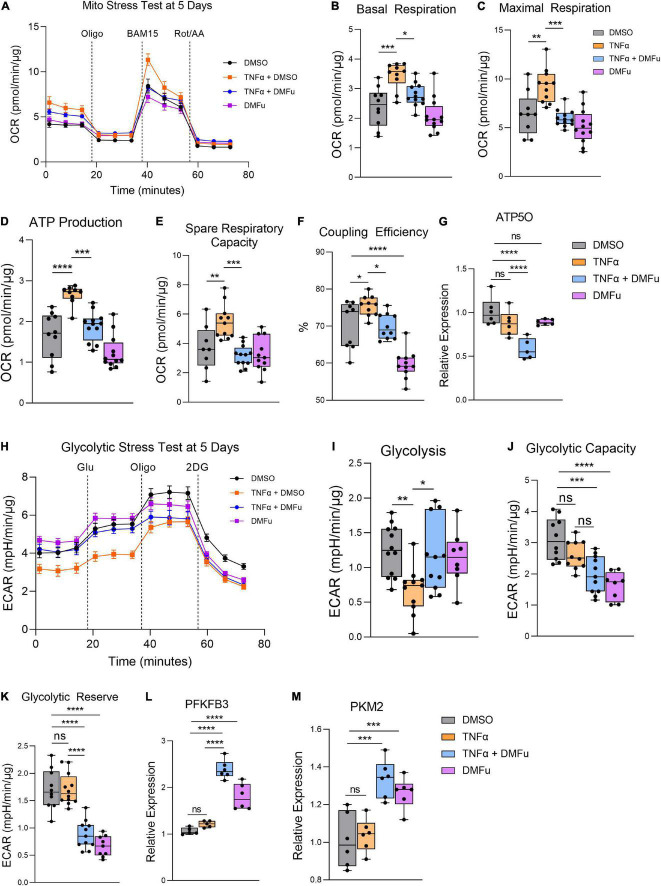
Pre-treatment with dimethyl fumarate (DMFu) blocks TNFα-induced metabolic reprogramming. **(A)** Real-time measurement of oxygen consumption rate (OCR) using the Seahorse XFe96 BioAnalyzer to assess OXPHOS parameters: **(B)** basal respiration, **(C)** maximal respiration, **(D)** ATP-linked respiration, **(E)** spare respiratory capacity, and **(F)** coupling efficiency based on responses to drug injections of oligomycin (Oligo), BAM15 and rotenone and antimycin A (Rot/AA) at 5 days with and without TNFα and/or DMFu or the vehicle control (DMSO) (*n* = 10–12, unpaired *t*-test). qPCR gene expression of **(G)** ATP5O following 24 h of treatments. **(H)** Real-time measurement of extracellular acidification rate (ECAR) using the Seahorse XFe96 BioAnalyzer to assess glycolytic function parameters including **(I)** glycolysis, **(J)** glycolytic capacity, and **(K)** glycolytic reserve based on responses to drug injections of glucose (Glu), oligomycin (Oligo), and 2-deoxyglucose (2DG) at 5 days of treatments (*n* = 10–12, unpaired *t*-test). qPCR gene expression of **(L)** PFKFB3 and **(M)** PKM2 following 24 h of treatments. *n* = 6 for gene expression, one-way ANOVA with Tukey’s *post hoc* analysis. Error bars are means ± SEM. * *p* ≤ 0.05; ** *p* ≤ 0.01; *** *p* ≤ 0.001; **** *p* ≤ 0.0001; ns, not significant.

On the Seahorse Mito Stress Test, DMFu blocked the elevated mitochondrial respiration induced by TNFα (Figure 6A). Specifically, DMFu blocked TNFα-dependent elevation of basal respiration (Figure 6B), maximal respiration (Figure 6C), ATP production (Figure 6D), spare respiratory capacity (Figure 6E), and coupling efficiency (Figure 6F). This blockade was accompanied by a reduction in gene expression of ATP5O, a key component of ATP synthase in the electron transport chain (Figure 6G). The Seahorse Glycolytic Stress Test showed that DMFu restored the suppression of glycolysis induced by TNFα (Figure 6H) with a statistically significant increase in glycolysis (Figure 6I), but no changes in glycolytic capacity (Figure 6J) and a reduction in glycolytic reserve (Figure 6K). The enhanced glycolysis with co-treatment of DMFu and TNFα is supported by the enhanced gene expression of glycolytic enzymes, namely, PFKFB3 (Figure 6L) and PKM2 (Figure 6M).

## Discussion

TNFα-induced inflammation in H-RPE is accompanied by a dramatic disruption in normal mitochondrial morphology and metabolic function. We show that TNFα induces the accumulation of engorged mitochondria with loss of cristae integrity and overactive metabolic function, promoting increased mitochondrial respiration, ATP production and reduced glycolysis in H-RPE. Pre-treatment of H-RPE with DMFu blocked TNFα-induced inflammation and normalized metabolic function and mitochondrial ultrastructural morphology.

An intricate interplay exists between inflammation and metabolic dysfunction at sites of ongoing inflammation (Kominsky et al., 2010). The enhanced OXPHOS and increased ATP production induced by TNFα in our study highlights the substantial bioenergetic challenge required to mount an inflammatory response in RPE. Intriguingly, our previous work showed that TGFβ2, an important cytokine in AMD pathogenesis, induces the opposite metabolic changes in RPE with reduced OXPHOS and enhanced glycolysis, leading to an overall reduction in ATP content (Shu et al., 2021). Since TGFβ2 itself does not induce inflammation, it is possible that the enhanced OXPHOS induced by TNFα may facilitate its pro-inflammatory activity. The capacity of RPE to switch between different bioenergetic profiles depending on cytokine stimulation shows the metabolic flexibility of RPE. Despite the capacity of both TGFβ2 and TNFα to induce robust EMT responses in RPE, a key component of pathogenic subretinal fibrosis in AMD (Shu et al., 2020, 2021), their divergent impact on metabolic reprogramming highlights the complexity of targeting metabolism as a therapeutic avenue for AMD. Future studies will continue to dissect the extensive crosstalk that exists between metabolism and EMT (Jia et al., 2021), examining how other metabolic pathways such as fatty acid oxidation and glutamine metabolism are impacted by TGFβ2 and TNFα in RPE.

Metabolic reprograming of immune cells during activation is highly cell-type and context dependent. Divergent metabolic profiles have been observed with B and T lymphocytes with B cells relying more on OXPHOS, while T cells rely more on aerobic glycolysis (Khalsa et al., 2019). Activation of B cells is bioenergetically demanding and consequently induces a progressive upregulation of OXPHOS to fulfill their new energy requirements (Price et al., 2018). Treatment of RPE with TNFα appears to mimic the metabolic changes observed in activated B cells. Indeed, activated B cells are known to secrete pro-inflammatory cytokines including TNFα and IL-6 (Gupta et al., 2013), which may, in turn, further potentiate inflammation and mitochondrial respiration. Our finding that TNFα increases OXPHOS in RPE has also been reported in platelets of aged mice where the pro-inflammatory activity of TNFα was linked to mitochondrial dysfunction, increased mitochondrial mass and metabolite rewiring to favor elevated pentose phosphate pathway intermediates (Davizon-Castillo et al., 2019). Future studies on comprehensive metabolomics analysis of TNFα-treated H-RPE will determine the precise metabolic pathway reconfigurations, further unraveling the complex interplay between inflammation and the RPE metabolome.

Activation of the alternative pathway of the complement cascade has been implicated in the pathogenesis of AMD and in particular, geographic atrophy (Desai and Dugel, 2022). Our study shows that TNFα robustly upregulates critical components of the complement cascade and that DMFu is able to suppress TNFα-induced complement activation. Intriguingly, the ability of DMFu to suppress complement activation is accompanied by enhanced angiogenic gene expression. VEGF-C, an established pro-angiogenic factor and enhancer of vascular permeability, was strongly induced by TNFα and further upregulated in the presence of DMFu, indicating a potential risk for promoting an angiogenic response as observed in neovascular AMD. Complement inhibition is believed to induce a shift in macrophage polarization from the pro-inflammatory M1 to the pro-angiogenic M2 macrophages (Cao et al., 2011). Indeed, in the clinical trial for the C3 inhibitor, APL-2, 18% of patients on monthly injections showed conversion to neovascular AMD, highlighting a critical link between complement inhibition and the risk of increased angiogenic potential (Park et al., 2019).

Our study highlights DMFu as a novel immunometabolic regulator of RPE. We show that the anti-inflammatory effect of DMFu was accompanied by a normalization of RPE metabolic function, blocking TNFα-induced elevated OCR and maintaining glycolysis at basal levels. The anti-inflammatory capacity of DMFu showed beneficial effects in an autoimmune uveitis rat model (Labsi et al., 2021) and optic neuritis murine model (Zyla et al., 2019). Further, DMFu promotes the survival of retinal ganglion cells (RGCs) after optic nerve crush (Mori et al., 2020) and protects against retinal degeneration in a light-induced photoreceptor loss mouse model (Dietrich et al., 2020). Mechanistically, studies have shown that DMFu activates Nrf2/heme oxygenase-1 (HO-1) (Zyla et al., 2019; Mori et al., 2020) and increases glutathione (Nelson et al., 1999; Dietrich et al., 2020) to mediate its anti-inflammatory and anti-oxidative effects. Intriguingly, in this study, DMFu alone induces significant metabolic changes compared to DMSO-treated control cells. For example, DMFu slightly reduces basal levels of PGC-1α gene expression and increases the gene expression of glycolytic enzymes, PFKFB3 and PKM2, despite reducing glycolytic capacity and reserve. These metabolic changes do not impinge on the potent anti-inflammatory activity of DMFu against TNFα in RPE. The favorable efficacy and safety profiles of oral administration of DMFu for psoriasis and multiple sclerosis highlights its promise as a therapeutic avenue for immunomodulation of retinal diseases, including AMD. Furthermore, DMFu will only be considered as a treatment against active pathology rather than as a prophylactic therapy.

Ultrastructural mitochondrial imaging on TEM revealed dramatic mitochondrial defects following TNFα treatment of H-RPE, which was prevented by co-treatment with DMFu. Typically, long fused mitochondria are associated with enhanced OXPHOS capacity compared to smaller, fragmented mitochondria with reduced OXPHOS (Galloway et al., 2012). Indeed, our data shows that TNFα-induced elevated OXPHOS is associated with enlarged mitochondrial size, highlighting the morphological plasticity of RPE and reciprocal relationship between bioenergetic status and mitochondrial morphology. Elevated OXPHOS levels may generate more reactive oxygen species (ROS) byproducts that can cause further oxidative damage to mitochondrial morphology (Jassim et al., 2021). It is hypothesized that increased oxidative damage to mitochondria may lead to increased mtDNA copy number as an initial compensatory mechanism (Wang et al., 2014). Our data shows that TNFα increases mtDNA copy number in RPE, which can be blocked by co-treatment with DMFu. Similar results were observed in a clinical study of obese diabetic participants where increased body mass index (BMI) was associated with increased TNFα production, inflammation and mtDNA copy number (Skuratovskaia et al., 2019).

Our study reveals the dynamic interplay between inflammation and metabolic function in RPE, highlighting the morphological and bioenergetic alterations in mitochondria following TNFα stimulation. TNFα-driven inflammation causes a dramatic increase in energy demands in RPE, leading to increased oxygen consumption and ATP production at the expense of mitochondrial health. Such pro-inflammatory activation of RPE *in vivo* would undoubtedly cause an imbalance in the tightly regulated metabolic ecosystem of the retina (Kanow et al., 2017) through altered nutrient and oxygen availability for neighboring retinal cells. DMFu effectively abolished the drastic mitochondrial changes induced by TNFα, maintaining normal mitochondrial morphology and bioenergetic profiles and thus serves as a promising therapeutic avenue for combating inflammation in AMD.

## Data Availability Statement

The original contributions presented in this study are included in the article/supplementary material, further inquiries can be directed to the corresponding author.

## Author Contributions

DYS and MS-G: conceptualization and design. DYS, MS-G, SIF, and TCF: methodology. DYS, SIF, and MMK: writing–original draft preparation. DYS, SIF, TCF, ERB, and EN-J: formal analysis and investigation. DYS, SIF, TCF, MMK, ERB, EN-J, LAK, and MS-G: writing–review and editing. DYS, MS-G, and LAK: project administration and funding acquisition. All authors contributed to the article and approved the submitted version.

## Conflict of Interest

The authors declare that the research was conducted in the absence of any commercial or financial relationships that could be construed as a potential conflict of interest.

## Publisher’s Note

All claims expressed in this article are solely those of the authors and do not necessarily represent those of their affiliated organizations, or those of the publisher, the editors and the reviewers. Any product that may be evaluated in this article, or claim that may be made by its manufacturer, is not guaranteed or endorsed by the publisher.
